# Evaluation of Some Benzo[g]Quinazoline Derivatives as Antiviral Agents against Human Rotavirus Wa Strain: Biological Screening and Docking Study

**DOI:** 10.3390/cimb45030156

**Published:** 2023-03-14

**Authors:** Hatem A. Abuelizz, Ahmed H. Bakheit, Mohamed Marzouk, Waled M. El-Senousy, Mohamed M. Abdellatif, Gamal A. E. Mostafa, Rashad Al-Salahi

**Affiliations:** 1 Department of Pharmaceutical Chemistry, College of Pharmacy, King Saud University, Riyadh 11451, Saudi Arabia; 2 Chemistry of Tanning Materials and Leather Technology Department, Organic Chemicals Industries Division, National Research Centre, Dokki, Cairo 12622, Egypt; 3 Food Environmental Virology Laboratory, Water Pollution Research Department, Environment and Climate Change Research Institute and Food-Borne Viruses Group, Centre of Excellence for Advanced Sciences, National Research Centre (NRC), 33 El-Buhouth Street, Dokki, Giza 12622, Egypt; 4 Department of Chemistry, Graduate School of Science, Tokyo Metropolitan University, 1-1 Minami Osawa, Tokyo 192-0397, Japan

**Keywords:** benzo[g]quinazolines, rotavirus Wa strain, docking study, cytotoxicity

## Abstract

Globally, rotavirus (RV) is the most common cause of acute gastroenteritis in infants and toddlers; however, there are currently no agents available that are tailored to treat rotavirus infection in particular. Improved and widespread immunization programs are being implemented worldwide to reduce rotavirus morbidity and mortality. Despite certain immunizations, there are no licensed antivirals that can attack rotavirus in hosts. Benzoquinazolines, chemical components synthesized in our laboratory, were developed as antiviral agents, and showed good activity against herpes simplex, coxsackievirus B4 and hepatitis A and C. In this research project, an in vitro investigation of the effectiveness of benzoquinazoline derivatives **1**–**16** against human rotavirus Wa strains was carried out. All compounds exhibited antiviral activity, however compounds **1**–**3**, **9** and **16** showed the greatest activity (reduction percentages ranged from 50 to 66%). In-silico molecular docking of highly active compounds, which were selected after studying the biological activity of all investigated of benzo[g]quinazolines compounds, was implemented into the protein’s putative binding site to establish an optimal orientation for binding. As a result, compounds **1**, **3**, **9**, and **16** are promising anti-rotavirus Wa strains that lead with Outer Capsid protein VP4 inhibition.

## 1. Introduction

Rotavirus (RV) is a non-enveloped double-stranded RNA virus that has eleven viral genes that code for a total of twelve proteins. Of these proteins, six are structural (VP1-4, VP6, and VP7) and the remaining six are non-structural (NSP1-6) [1,2]. The antigenicity of the VP6 protein and the RNA can be used to classify rotaviruses into groups A–G. All three of the A, B, and C classes are capable of infecting both humans and other animals [3,4]. Transfer of RV through the fecal-oral channel results in fast replication of the virus in the cells that line the small intestine. This results in severe diarrhea and dehydration in infants and young children all over the world. Despite a significant amount of research using animal models, the pathophysiology of rotavirus is not well understood [5,6,7]. The pathophysiology of a rotavirus infection includes the death of enterocytes, alterations in fluid balance, and local villous ischemia that causes vascular injury; however, none of these mechanisms may explain diarrhea in the absence of histopathologic abnormalities. There is a connection between the NSP4 (nonstructural protein) gene and the virulence of the rotavirus [7]. This was demonstrated by both virulent and attenuated porcine and other rotaviruses, as well as gnotobiotic (Gn) pigs. The membrane instability and Ca^2+^ transport modulation that are caused by the viral enterotoxin NSP4. When exposed to a severe strain of rotavirus, infant mice that had been passively vaccinated with NSP4 antibodies experienced significantly less diarrhea. Antibodies against NSP4 may decrease the severity of diarrhea caused by viruses [8]. Several studies investigated whether virulent or attenuated Wa HRV (resembling natural infection or live oral vaccines, respectively) would induce systemic and intestinal antibody responses to NSP4, as well as whether there was a correlation between antibody responses to Wa NSP4 and diarrhea in Gn pigs that had been challenged with virulent Wa HRV [8]. In the context of secretory diarrhea induced by rotavirus, it is possible for an excessive amount of fluid and electrolyte secretion to actually happen into the lumen of the digestive tract. The Na^+^/H^+^ exchanger (NHE) and other Na^+^-coupled transport mechanisms at the luminal membrane of the small intestine are responsible for the fluid absorption that actually occurs in this system. Additionally, the NHE’s protein expression was validated through the use of immunocytochemistry [9,10,11]. In addition, the presence of calcium ions is a significant factor that contributes to the enhancement of the rotavirus infection process. It has been discovered as a critical component in both the activation of the endogenous transcriptase of rotavirus and the maintenance of capsid integrity [12]. Ions of calcium play an essential part in the progression of RV infection throughout the body, from cell invasion and transcriptional activation to morphogenesis and cellular lysis, viral particle release, and the subsequent behavior of infected proteins. In human colon adenocarcinoma (Caco-2) cells, the RV-Wa has also been shown to be capable of preventing the expression of calmodulin kinase II (CaMKII) and calmodulin (CaM) [5]. Wakabayashi and colleagues have demonstrated that changes in intracellular calcium concentration can cause CaM conformation changes, which in turn activate CaMKII and further phosphorylate serine and/or threonine residues of NHEs. This demonstrates the significance of calcium in the NHE regulatory pathway and highlights the significance of calcium in the NHE regulatory pathway [13]. Taking into consideration the information presented above, rotavirus may reduce NHE expression by inhibiting CaM expression, which in turn decreases intracellular calcium levels, and by subsequently reducing NHE phosphorylation and expression, which in turn suppresses CaMKII activity. Both effects are thought to be responsible for the reduction. Even though RV-induced changes in intracellular calcium can have an effect on CaM expression, the particular mechanism by which this happens still needs a lot of research. The creation of a vaccine against rotavirus should be a top WHO goal. According to the Global Alliance for Vaccines and Immunization (GAVI), vaccination can reduce the number of deaths caused by rotavirus by 2.46 million between the years 2007 and 2025 in areas of the world where the vaccine is already in use [1,2]. However, the development of a vaccine is impossible in the absence of a reliable system for the collection of vaccine materials from either humans, animals, or the environment. The implementation of rotavirus concentration strategies might prove useful in this scenario. As a direct consequence of this, innovative strategies for preventing and treating diarrhea and gastroenteritis brought on by rotaviruses are urgently required. Numerous different chemicals, both those that exist naturally and those that are created, have been shown to be capable of inhibiting the replication of the rotavirus. In MA104 cells, the synthetic compounds 1-3-d-ribofuranosyl-1,2,4-triazole-3-carboxamide (ribavirin), 3-deazaguanine (3-DG), and inosine pranobex were found to have an inhibitory effect against the SA11 strain of simian rotavirus [14,15]. It has been demonstrated that black tea (theafavin), *Stevia rebaudiana* (anionic polysaccharide component), Brazilian medical plants (*Quillaja saponaria*), and nutritional plants can all inhibit the uptake or replication of rotaviruses in humans and animals [16,17,18,19]. It was unreasonably upbeat to believe that contagious viral and bacterial diseases will be eradicated in the not-too-distant future as a direct result of the discovery and development of novel drugs. However, as a response to the discovery of antimicrobial drugs, there has been an increase in the prevalence of drug-resistant microorganisms, which continue to cause problems for humans. There is currently no treatment that is proven to be effective for rotavirus gastroenteritis. Rehydration through oral administration has proven to be the most successful treatment for mitigating the consequences of infection. To treat rotavirus gastroenteritis, however, mucosal and systemic immunomodulatory drugs, such as active antiviral drugs, are frequently insufficient [6].

**Figure 1:**
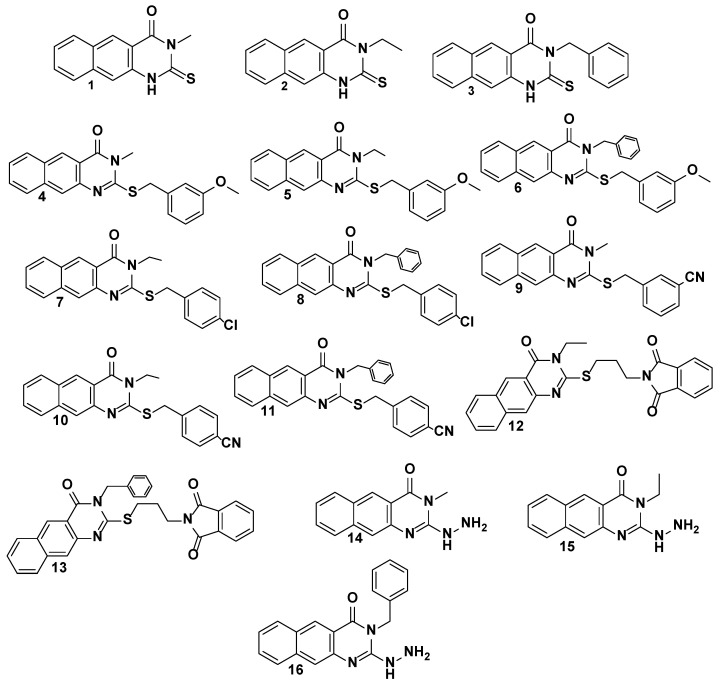
The target benzo[g]quinazolines **1**–**16**.

Benzoquinazolines, the basic components of numerous well-known heterocyclic systems, have played a significant role in the creation of various bioactive molecules. Extensive research conducted on benzoquinazolines in our lab has demonstrated their powerful pharmacological effects in a variety of contexts such as antioxidant, antimicrobial, anticancer and antidiabetic agents [20,21,22,23,24,25,26,27,28,29], and particularly against a number of different viral infections as HSV-1, HSV-2, HAV, HCV and coxsackievirus B4 [30,31,32]. Through the analysis of our data, we were able to gather insights into the characteristics that will define future trials, which paved the path for the development of benzo[g]quinazolines as anti-human rotavirus Wa strain drugs. Consequently, in the current investigation, we evaluated a new group of 2-thioxo-benzo[g]quinazolines **1**–**16** (Figure 1) to determine their effectiveness against human rotavirus infection. To learn more about how small molecules behave in the binding sites of target proteins and to understand basic biochemical processes, we used molecular docking with MOE 2015.10 software to look at how compounds **1**, **3**, **9**, and **16** interact with the active site of the human rotavirus strain Wa protein.

## 2. Materials and Methods

### 2.1. The Target 2-Thioxo-Benzo[g]quinazoline (1–16)

All compounds **1**–**16** were previously synthesized and fully characterized [25,27,31]. Analytical data such as NMR, IR, and MS spectra were used to completely establish and confirm the structures of compounds **1**–**16**. It was feasible to obtain the benzoquinazolines **1**–**16** in the form of solid colored materials. In a brief, the parent intermediates **1**–**3** can be obtained in good yields by reacting ethyl(methyl)isothiocyanate or benzylisothiocyanate with 3-amino-2-naphthaoic acid in a basic medium using trimethylamine while the reaction was carried out under a refluxing condition in dimethyl formamide (DMF) for 3–5 h. The thioalkylated benzo[g]quinazolines **4**–**13** were produced in good to high yields after compounds **1**–**3** were subjected to treatment with the appropriate aralkyl halide (benzyl substituted halides) or hetroalkyl in the presence of a base as potassium carbonate (K_2_CO_3_) at 80 °C for 20 h. The respective **14**–**16** could be obtained by hydrazinolyzing products **1**–**3** in boiling DMF for 15–18 h. The details of their chemical and physical properties are reported [25,27,31].

### 2.2. Cytotoxicity Test

It was carried out in accordance with the methods that were reported [33,34]. In 1 mL of ethyl alcohol, 100 mg of sample was dissolved. A sample of 24 μL of 100× antibiotic–antimycotic mixture was added to 1 mL of sample to decontaminate it. Then, bi-fold dilutions were done to 100 μL of the original dissolved sample, and 100 μL of each dilution was inoculated in Hep-2, BGM, MA104, and CaCo-2 cell lines (obtained from the Holding Company for Biological Products & Vaccines VACSERA, Giza, Egypt) previously cultured in 96 multi well plates (Greiner-Bio one, Frickenhausen, Germany) to estimate the sample’s non-toxic dose. Inverted light microscope cell morphology and trypan blue dye exclusion cell viability tests were used for cytotoxicity assay.

### 2.3. Cell Morphology Evaluation by Inverted Light Microscopy

The Hep-2, BGM, MA104, and CaCo-2 cell cultures were each seeded at a concentration of 2 × 105 cells/mL and grown individually in 96-well tissue culture plates (Greiner-Bio one, Frickenhausen, Germany). After an incubation period of 24 h at 37 °C in an atmosphere containing 5% (*v*/*v*) CO_2_, cell monolayers were confluent. At this point, the media was withdrawn from each well and replaced with 100 μL of bi-fold dilutions of the sample examined that were prepared in Dulbecco’s Modified Eagle Medium (DMEM) (GIBCO BRL, Darmstadt, Germany). For the purpose of cell controls, 100 μL of DMEM containing no samples was added. All the cultures were kept in an atmosphere with 5% (*v*/*v*) CO_2_ that was humidified and incubated at 37 °C for a total of 72 h. Daily observations showed that the cells had lost their confluence, were rounding and shrinking, and that the cytoplasm was granulating and vacuolizing. Morphology shifts were observed and scored [33].

### 2.4. Cell Viability Assay

Trypan blue dye was excluded from the experiment [34]. Trypan blue dye was excluded from the experiment [35]. In 12-well tissue culture plates, Hep-2, BGM, MA104, and CaCo-2 cells (2 × 10^5^ cells/mL) were cultivated (Greiner-Bio one, Frickenhausen, Germany). Following an incubation period of 24 h, 100 μL of tested sample dilutions, also known as bi-fold dilutions, were added to each well, and the results were examined in the same manner as described previously. After 72 h, the medium was removed, the cells were trypsinized, and an equal amount of an aqueous solution containing 0.4% (*w*/*v*) trypan blue dye was added. Counting the number of viable cells required phase contrast microscopes.

### 2.5. Determination of Rotavirus Titers Using Plaque Assay

The Rotavirus Wa strain, at a concentration of 1 × 10^7^ PFU/mL, was mixed with non-toxic dilutions in a volume of 100 μL. At a temperature of 37 °C and 10 μg/mL of trypsin, the rotavirus stocks became infectious after 30 min. The mixture was incubated for half an hour at 37 °C. The Hep 2, BGM, and MA104 cell lines were seeded into the wells of 12 multi-well plates and then injected with 100 μL (10-fold) dilutions of the treated and untreated rotavirus Wa strain after an hour of adsorption at 37 °C in an atmosphere containing 5% CO_2_-water vapor without shaking. The plates were bounced at regular intervals to prevent the cells from drying out. Following the adsorption step, 1 mL of a 2× media consisting of Dulbecco’s Modified Eagle Medium, Gibco-BRL (DMEM), and 1% agarose were added to each well. The mixture of medium and agarose was given an additional 0.5 μg/mL of trypsin so that it could test for the rotavirus Wa strain. The plates were kept in an incubator with an environment containing 5% CO_2_ and water vapor at 37 °C. After being fixed with formalin and stained with crystal violet at a concentration of 0.4%, the plaques were counted. After that, the PFU/mL viral titers were determined [35].

### 2.6. Molecular Docking

The Molecular Operating Environment (MOE) was created with the intention of studying molecular docking and 3D/2D structures [20,31,36]. We used the MOE-Dock docking program, and the MOE-implemented ligplot to visualize the protein-ligand interaction. The active site of the Human rotavirus strain Wa protein, which serves as the target, was specified using a co-crystallized ligand atom. This ligand atom is in the pocket (the latter will be ignored during docking). The chemical structures of ligand, substructures, and reactions were drawn, displayed, and characterized using Marvin Sketch software (Budapest, Hungary) (version 21.19.0 (internal build ID: 21.19.0-13698)) and ChemAxon (Budapest, Hungary) (https://www.Chemaxon.com accessed on 10 February 2023).

### 2.7. Statistics

Mean and standard deviation were calculated.

## 3. Results and Discussions

### 3.1. Biological Evaluation

**Figure 2:**
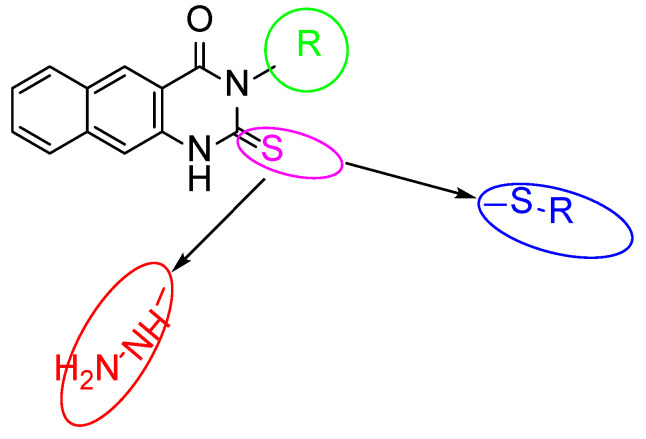
Structural modification on benzoquinazolines. Increase in the activity accompanied by inserting methyl group at position 3; Replacing C = S by S-Bn substituted as in case compound **9** resulted in increasing the activity; Replacing C = S by -NH-NH_2_ as in case compound **16** resulted in increasing the activity in regards to compounds **14** and **15**; Presence of electron donating/withdrawing groups produced a variety of activity as in **8, 6** and **9**.

Benzo[g]quinazolines have been found to be effective against hepatitis A and C viruses (HAV and HCV), herpes simplex virus (HSV1 and 2), and coxsackievirus 4 (CVB4). Due to their much greater activity and low cytotoxicity, benzo[g]quinazoline derivatives have been prioritized as a novel class of potent antiviral substances against the rotavirus strain in our ongoing research. This study investigated 2-thioxo-benzo[g]quinazolines (Table 1 and Table 2) against human rotavirus Wa strain in vitro. In the experimental part, the target benzo[g]quinazolines (**1**–**16**) were evaluated for cytotoxicity in Hep2, BGM, and MA104 cells (Table 1). Targets **1**–**16**’s nontoxic dosages, 25–75 g/mL, were comparable. Cytotoxicity experiments showed that Hep-2 and MA104 cells were more resistant to benzoquinazoline toxicity than BGM cells. Higher concentrations of **1**–**16** did not cause cell death, rounding, or shrinkage in Hep-2 and MA104 cells. The various cell lines’ type, origin, structure, and appearance may clarify this. The BGM, Hep-2, and MA104 cells may resist toxic compounds due to their differences. Most benzoquinazolines showed antiviral effects, but their efficacy varied. Due to the unique virus structure, size, and genetics, this is typical. In spite of the RNA genomic structure of rotavirus, benzoquinazolines have been shown to cause a variety of responses. Different quantities and types of viral RNAs are capable of inducing a variety of antiviral bioassay responses from the test compound. When determining the level of viral infectivity, it is probable that only the compound’s direct action on the virus was taken into consideration, and not its potential to stop viral adsorption or interrupt the cycle of viral replication. The investigations only target the virus with non-toxic quantities of the drugs that are being evaluated, which may explain why this is the case. The results of this study could have been altered by the drugs that were assessed at non-toxic doses yet still had an effect on the genome. A further explanation is that higher doses may increase the probability of an antiviral impact, as demonstrated by the fact that different cell lines showed no toxicity at differing levels. This explanation is supported by the fact that the higher doses were assayed at. All the tested compounds **1**–**16**, displayed varying degrees against human rotavirus Wa activity, ranging from low to high (Table 2); however, compounds **4**, **5**, **7**, **12**, and **15** showed the lowest activity (10–16%). With reduction percentages ranging from 50 to 66%, compounds **1**, **2**, **3**, **9**, and **16** show good antiviral activity against the human rotavirus Wa strain. On the other hand, benzoquinazolines **8**, **10**, **11**, and **14** showed only moderate efficacy (20–30%). As was mentioned before, the pharmacological effects of substitutions made at positions 2 and 3 on the benzoquinazoline nucleus are distinct from one another. Increased inhibitory effects can be achieved by making substitutions at position 3 with either an aliphatic group (methyl and ethyl) or an aromatic moiety (benzyl) (Figure 2). However, positive outcomes can be achieved by attempting to make substitutions at position 2 with a benzyl bearing electron donating/withdrawing groups as methoxy, Cl and CN. Using the findings described above, a preliminary SAR was constructed. According to the results of Table 2, the parent compounds (**1**, **2**, and **3**) had the highest activity (50–60%) against the Human rotavirus Wa strain. Compound **1**, which has a methyl substitution at position 3 in the benzo[g]quinazoline skeleton, displaying the highest reduction (60%), followed by compounds **3** and **2**, which contain benzyl and ethyl groups, respectively. The improvement in the activity profile is achieved through a chemical conversion of the **1**’s thioxo group into the **9**’s thioether group (66%). However, it was unfavorable for parents **2** and **3**. Compounds with substituted methoxy groups **4**–**6** exhibited distinct biological effects. Compound **6** with a *N*-benzyl substitution was shown to have a higher level of activity compared to compounds **4** and **5** with *N*-methyl and *N*-ethyl substitutions, lending credence to the concept that lipophilicity is a factor. Compound **8**, which had a chloro substituted benzyl group, was more active than compound **7**, as well as supporting the lipophilicity fact. Compounds **10** and **11** with a cyano substituent in the para position are not as effective as benzoquinazoline **9**, which contains a cyano substituent in the meta position of the benzyl moiety, in combating HRV. Compounds **10** and **11** displayed the same behavior, which provides further evidence for the lipophilicity point that was just discussed**.** Derivatives **14**–**16** are generated by performing a substitution in which a thioxo functional group is replaced to a hydrazine functional group. Only derivative **16** shown increased activity (50%) in this study. Therefore, inserting a hydrogen-rich group or adding an electron-donating or electron-withdrawing group like methoxy, chloride, or cyano into the benzyl group produced a variety of activity patterns.

### 3.2. Docking Study

**Figure 3:**
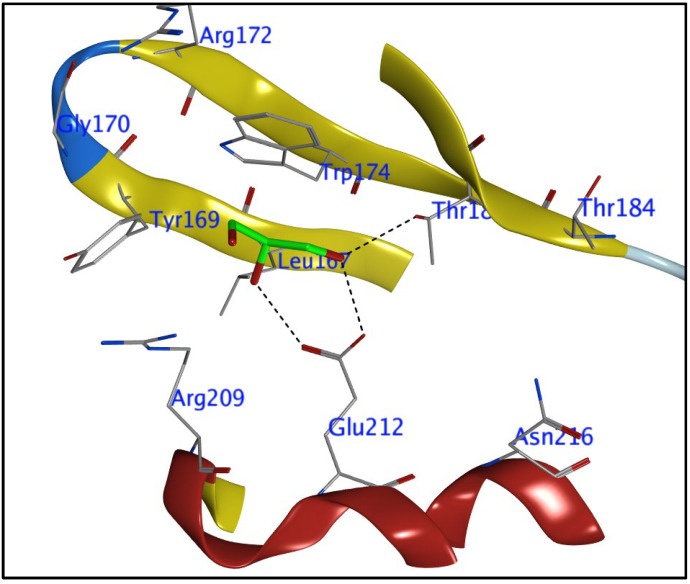
Predicted binding pocket of the Human rotavirus strain Wa by MOE-2015.10 a co-crystallized ligand atom that is present in the pocket.

To identify the location of the active site, an atom from a co-crystallized ligand that was discovered in the pocket of the Human rotavirus strain Wa protein (target) was employed (that ligand was ignored during docking). After determining the places in which the docking computation for molecules needed to take place, the locations of the co-crystallized ligand atoms are illustrated in Figure 3. Because of the significant levels of biological activity exhibited by the compounds **1**, **3**, **9**, and 16 these four were selected for the study of molecular docking with the human rotavirus Wa strain. All these compounds were initially designed in Marvin Sketch, exported to mol files, and then energy minimized in MOE with the default settings (Marvin was used for drawing, displaying, and characterizing chemical structures, substructures, and reactions, Marvin version 21.19.0 (internal build ID: 21.19.0-13698), ChemAxon (https://www.Chemaxon.com accessed on 10 February 2023).

Determining the ligand-target protein binding interaction in the human rotavirus Wa strain was the goal of this particular study. The nature of the binding interactions was investigated using molecular docking, with the MOE-default dock’s settings serving as the default for the tools. The ligand-bound target site was visible in the crystal structure of the rotavirus. Crystallographic analysis of the human rotavirus combined with the co-crystallized ligand of the ligand-binding site revealed the presence of the amino acids Arg172, Gly170, Trp174, Thr184, Tyr169, Arg209, Glu212, Asn216, and Leu197. The active site of the human rotavirus strain Wa protein (target) was specified using a co-crystallized ligand atom that is present in the pocket (the latter will be ignored during docking) (Figure 3) [37].

Four of the most effective compounds (**1**, **3**, **9**, and **16**) were analyzed for their docking scores and binding interactions with the target human rotavirus protein, and the results are summarized in Table 3.

**Figure 4:**
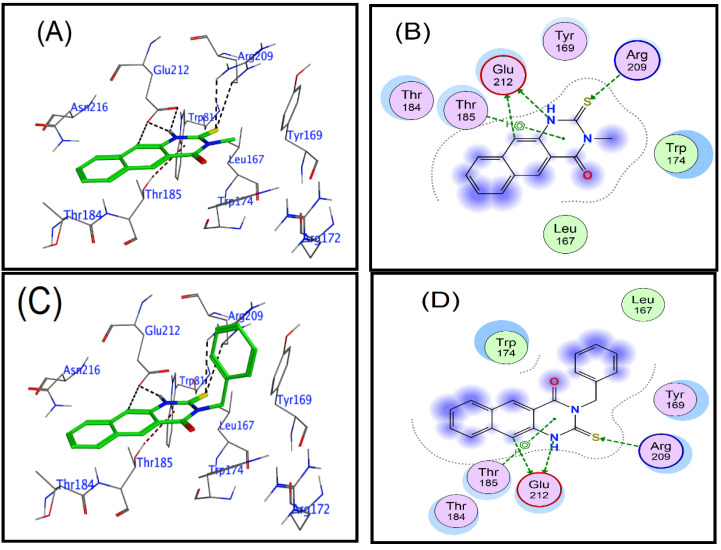

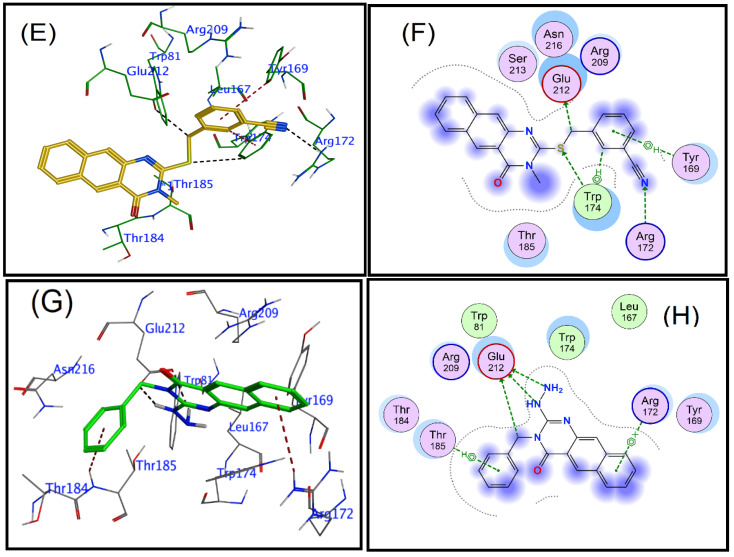
Human rotavirus ligand interaction diagram (2DWR). Active site residues: polar residues pink, hydrophobic residues green, acidic residues red, basic residues blue. Green and blue arrows represent hydrogen bonding to sidechain and backbone atoms. (**A**,**B**): 2D and 3D for interactions of compound **1;** (**C**,**D**): 2D and 3D for interactions of compound **3;** (**E**,**F**): 2D and 3D for interactions of compound **9;** (**G**,**H**): 2D and 3D for interactions of compound **16**.

The benzoquinazolines **1**, **3**, **9**, and **16** demonstrated the best reduction efficacy against human rotavirus (Table 2), and these compounds also showed good interaction with the target protein, as evidenced by their docking scores (−3.95, −4.21, −4.83, and −4.29, respectively), which are given in Table 3. The ligand-receptor interaction occurs in both three dimensions and two dimensions (Figure 4). The selected compounds (**1**, **3**, **9**, and **16**), the human rotavirus Wa were placed into the pocket created by Trp174, Thr184, and Thr185, Arg209, and Glu212, Ser213, and Asn216, and it appeared to be stabilized through hydrogen bonding and hydrophobic interactions. Compound **9** was proven to be the most efficient inhibitor when bound to the human rotavirus strain Wa. The amino acids Glu212, Trp174, and Arg172 contributed to the formation of hydrogen bonds with the aliphatic carbon of the benzene group, thiol group, and nitrile group, respectively. In addition to this, the arene ring established an interaction with Tyr169 and Trp174 through the route of π-H. The thiol and aromatic amine groups of benzoquinazolines **1** and **3** created hydrogen bonds with Arg209 and Glu212, respectively, while benzoquinazolines **1** and **3** were simultaneously bonding to the human rotavirus strain Wa. In addition to this, the arene ring established a pi-H interaction with the Thr185. Whereas compound **16** forms three hydrogen bonds, two by the hydrazine group with Glu212 and the third with linked carbon also with Glu212, in addition, compound **16** forms two π-bond by the benzene and arene rings in benzoquinazolines with Thr185 and Arg172, respectively (Figure 4).

**Figure 5:**
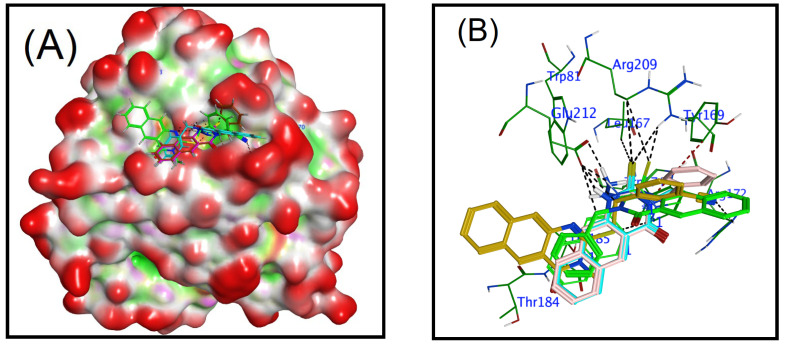
Interactions between ligands and proteins. (**A**) Surface representation of the Human rotavirus strain Wa (PDB ID: 2DWR), in three-dimensional representation, with an overlay of studied compounds (**1**, **3**, **9**, and **16**) at the active site of the enzyme (polar, hydrophobic, and exposed surfaces colored as pink, green, and red, respectively); (**B**) Predicted binding poses of docked ligands (sticks) with an overlay, only residues located within a 5Å radius of bound ligands are displayed (lines). The hydrogen bond is shown by black dashed lines.

An overlay of compounds **1**, **3**, **9**, and **16** at the binding site of the human rotavirus strain Wa is shown in Figure 5A. The (PDB ID: 2DWR) in all investigated compounds occupied a close position in the pocket of the enzyme, whereas Figure 5B displays the overlay of ligands in the active site of the enzyme, and demonstrates the position of ligands in the active site that interact with crucial residues.

## 4. Conclusions

Benzo[g]quinazolines **1**–**16** were investigated for both their antiviral activity against the human rotavirus Wa strain and their cytotoxicity in the cell lines Hep2, BGM, and MA104. The benzoquinazolines **1**, **3**, and **9** demonstrated the most effective reducing activity against the Wa strain of human rotavirus. Benzoquinazolines **1**, **3**, **9**, and **16** are superimposed on a schematic of the binding site for human rotavirus strain Wa. All the compounds examined fit into the protein compartment in close proximity to one another (PDB ID: 2DWR). The ligand overlay at the active site of the protein is depicted, demonstrating the locations of the ligands that facilitate interactions between the protein and crucial residues. Insights into the properties required for optimal anchoring of the target compounds to limit virus plaque formation were obtained through structure-activity relationships study of the targets. Substitutions at either position 2 or 3 on the benzoquinazoline nucleus have different pharmacological effects. Effectiveness can be improved by inserting at position 2 using benzyl substituted groups that donate or accept electrons. This was indicated by compound **9**, which has appeared to possess the best activity. In addition, the positioning of substituted groups in benzyl has been found to cause a variety of antiviral effects, as demonstrated by **9**, **10**, and **11**; similarly, the behavior demonstrated by compounds **7** and **8** has been shown to be driven by the same approach. These insights could be taken into consideration when developing new antiviral treatments. Benzoquinazolines’ mechanism of action can only be fully comprehended via the completion of additional research in the laboratory. Additional research is required to investigate the antiviral efficacy of the constituents of the various promising materials in order to identify the particular significant portion that is accountable for the antiviral potency or to verify that the synergism of the fractions is responsible for the antiviral efficacy of the materials that have been tested.

## Figures and Tables

**Table 1:** Non-toxic doses of investigated benzoquinazolines (**1**–**16**) on various cell lines.

Comp.	Non-Toxic Doses (µg/mL)		
	BGM	MA104	Hep-2
**1**	60	65	65
**2**	60	60	60
**3**	60	75	70
**4**	50	55	55
**5**	55	55	60
**6**	65	65	65
**7**	60	60	60
**8**	50	50	50
**9**	65	75	70
**10**	50	55	50
**11**	65	65	65
**12**	25	30	30
**13**	40	50	45
**14**	55	60	60
**15**	55	55	55
**16**	60	60	65

**Table 2:** Rotavirus Wa strain reduction percentages by non-toxic doses **1**–**16**; three results against three doses of virus (1 × 10^7^) PFU/mL are Mean ± SD.

Compound	% Reduction	Compound	% Reduction
**1**	60% ± 3%	**2**	50% ± 3%
**3**	53.3% ± 3%	**4**	10% ± 3%
**5**	10% ± 4%	**6**	20% ± 4%
**7**	16.7% ± 3%	**8**	30% ± 3%
**9**	66.7% ± 1%	**10**	20% ± 4%
**11**	30% ± 2%	**12**	16.7% ± 2%
**13**	20% ± 3%	**14**	30% ± 3%
**15**	13.3% ± 3%	**16**	50% ± 3%

**Table 3:** Molecular docking interaction between selected compounds and target (the Human rotavirus strain Wa (PDB ID: 2DWR)).

Cp	Ligand		Receptor				Interaction	Distance	E (kcal/mol)	Score (kcal/mol)
**1**	N	9	OE1	GLU	212	(A)	H-donor	3.61	−0.3	−3.953
	N	9	OE2	GLU	212	(A)	H-donor	3.11	−5.2	
	C	25	OE2	GLU	212	(A)	H-donor	3.38	−0.3	
	S	27	CD	ARG	209	(A)	H-acceptor	3.71	−0.8	
	S	27	NH1	ARG	209	(A)	H-acceptor	3.57	−0.8	
	6-ring		OG1	THR	185	(A)	pi-H	4.2	−1.3	
**3**	N	19	OE2	GLU	212	(A)	H-donor	3.17	−4.3	−4.209
	C	35	OE2	GLU	212	(A)	H-donor	3.35	−0.3	
	S	37	CD	ARG	209	(A)	H-acceptor	3.86	−0.7	
	S	37	NH1	ARG	209	(A)	H-acceptor	3.37	−0.6	
	6-ring		OG1	THR	185	(A)	pi-H	4.1	−1.3	
**9**	C	10	OE2	GLU	212	(A)	H-donor	2.96	−0.7	−4.826
	S	9	CZ3	TRP	174	(A)	H-acceptor	4.4	−0.2	
	N	22	NH1	ARG	172	(A)	H-acceptor	3.84	−0.2	
	C	23	6-ring TRP		174	(A)	H-pi	4.53	−0.2	
	6-ring		CE2	TYR	169	(A)	pi-H	4.56	−0.2	
**16**	C	4	OE2	GLU	212	(A)	H-donor	3.44	−0.4	−4.287
	N	19	OE2	GLU	212	(A)	H-donor	2.91	−5	
	N	21	OE1	GLU	212	(A)	H-donor	3.33	−0.7	
	6-ring		CE2	TYR	169	(A)	pi-H	4.85	−0.2	
	6-ring		NH2	ARG	172	(A)	pi-cation	4.4	−0.9	

## Data Availability

Not applicable.
